# The Effects of Soil Moisture on the Pupation, Survival, and Emergence of the Tomato Leafminer, *Tuta absoluta*

**DOI:** 10.3390/insects17060603

**Published:** 2026-06-08

**Authors:** Jingxin Zhang, Xinyu Wang, Chunhong Yang, Yan Shi, Fanghao Wan, Bin Zhang

**Affiliations:** 1 Shandong Engineering Research Center for Environment-Friendly Agricultural Pest Management, China-Australia Joint Institute of Agricultural and Environmental Health, College of Plant Health and Medicine, Qingdao Agricultural University, Qingdao 266109, China; 2 Shenzhen Branch, Guangdong Laboratory for Lingnan Modern Agriculture, Genome Analysis Laboratory of the Ministry of Agriculture and Rural Affairs, Agricultural Genomics Institute at Shenzhen, Chinese Academy of Agricultural Sciences, Shenzhen 518000, China; 3 Department of Renewable Resources, University of Alberta, Edmonton, AB T6G 2E3, Canada

**Keywords:** *Tuta absoluta*, soil moisture, pupation, emergence, integrated pest management

## Abstract

The tomato leafminer, *Tuta absoluta* (Meyrick), is a globally invasive lepidopteran pest whose pupal stage commonly occurs within or at the soil surface. Soil moisture is a primary abiotic driver shaping soil microhabitats and therefore likely to influence pupation behavior, pupal survival and emergence. We quantified how six controlled soil moisture levels (0%, 20%, 40%, 60%, 80% and 100% saturation, wet-weight basis) affect pupation site selection, pupation depth, pupal survival and emergence under laboratory conditions, and performed a four-choice moisture preference assay. Larvae preferentially selected shallow soil layers (0–1 cm) and showed a marked behavioral preference for relatively low moisture (20–40%); maximum emergence (83%) occurred at 20% moisture. Both extreme drought (0%) and high saturation (≥80%) strongly reduced survival and emergence. These unimodal responses indicate that soil moisture constrains *T. absoluta* recruitment and may be exploited as a target for ecologically based control. The results provide a quantitative basis for soil-moisture-oriented IPM tactics and identify field validations and formulation/timing optimizations required to translate laboratory findings into practice.

## Simple Summary

The tomato leafminer is a major pest that damages tomato plants worldwide. Its young stage, the pupa, often lives in the soil. This study examined whether changing soil wetness could be used to control this pest. We tested how well pupae survive and turn into adult moths in soils ranging from completely dry to fully waterlogged. We found that the insects strongly preferred slightly dry soil. Most adult moths successfully emerged when soil moisture was around twenty to forty percent. However, in very dry or very wet soils, many pupae died. In waterlogged soil, all the pupae died within days. Most insects also stayed in the very top layer of soil. These findings suggest that farmers could help control this pest by briefly wetting the soil surface when the insect is entering the pupal stage. This method could reduce the need for chemical sprays, offering a more natural way to protect crops and support sustainable farming.

## 1. Introduction

The tomato leafminer, *Tuta absoluta* (Meyrick) (Lepidoptera: Gelechiidae), causes severe losses in tomato production globally and has rapidly spread beyond its native range in South America [1,2,3]. Larvae mine leaves, stems and fruits, and chemical control is complicated by the cryptic feeding habit and rapid resistance development, motivating integrated, ecology-based control strategies that exploit vulnerabilities across the pest’s life cycle [1,4,5,6]. The pupal stage which commonly occurs in the soil or at the soil–plant interface is one such vulnerability but has been relatively understudied with respect to abiotic microhabitat drivers such as soil moisture and texture. Quantifying how moisture affects pupation site selection, depth distribution, survival and emergence is therefore important for both fundamental population ecology and for designing novel IPM tactics that target soil stages.

Soil moisture can significantly influence pupal survival and emergence in soil-dwelling insects, although the effects may vary among species and conditions. For example, in *Helicoverpa punctigera* and *H. armigera*, soil moisture ranging from dry to very wet did not affect pupal survival or moth emergence when pupae burrowed into the soil; however, simulated rainfall after pupation reduced survival by disrupting emergence tunnels, an effect that was more pronounced when prepupae had burrowed into dry rather than moist soil [7]. In the leaf-cutter wasp *Priophorus fulvostigmatus*, soil moisture significantly affected pupation and emergence, with 8–16% moisture being most suitable, whereas water immersion of mature larvae in soil significantly reduced emergence rates [8]. Despite such species-specific variation, for many soil-dwelling insect stages, pupal survival exhibits a unimodal response to moisture: intermediate moisture supports development and emergence, while extremes (very dry or waterlogged) increase mortality [9]. Therefore, disentangling moisture effects is key to predicting field outcomes.

This study quantifies *T. absoluta* pupation responses across a moisture gradient and interprets the results to inform ecological, moisture-aware IPM strategies. We hypothesize (i) *T. absoluta* larvae will show behavioral preference for a limited moisture range associated with maximal pupal survival, (ii) pupation depth will vary with moisture, and (iii) both extreme desiccation and saturation will reduce pupal survival, creating opportunities for moisture-based control. The laboratory assays reported here test these hypotheses and are used to derive practical management recommendations and research priorities.

## 2. Materials and Methods

### 2.1. Insect Source and Laboratory Rearing

The populations of *T. absoluta* used in this study originated from the colony maintained at the Qingdao Agricultural University. The specimens were reared for multiple generations under controlled conditions (27 ± 1 °C; photoperiod L:D = 14:10; relative humidity 60 ± 10%). Adult moths were provided with a honey-sugar solution, and larvae were allowed to feed on detached tomato leaves prior to pupation. Rearing methods ensured the larvae entering the pupation assays were healthy, uniformly aged (fourth instar when used for pupation preference and depth assays), and had not been exposed to insecticides or experimental pathogens.

### 2.2. Soil Collection and Basic Properties

Soil was collected from the surface layer (0–10 cm) of an agricultural field on the campus of Qingdao Agricultural University. After air-drying, the soil was passed through a 2 mm sieve, mixed thoroughly, and stored for use. Soil texture was determined by the hydrometer method, yielding 52.3% sand, 32.0% silt, and 15.7% clay, which corresponds to a sandy loam according to the USDA soil texture classification. Soil pH was measured by the potentiometric method (soil:water = 1:2.5) and averaged 7.27.

### 2.3. Soil Moisture Treatments and Saturation Determination

Soil moisture was expressed as relative soil water content (RSWC, %), where 100% RSWC was defined as the maximum amount of water that the soil can retain after saturation and free drainage (i.e., capillary water-holding capacity). To determine this value, 100 g of oven-dried soil (105 °C for 24 h) was placed in a transparent cup with holes at the bottom. Distilled water was slowly added from the top until the soil was completely saturated, after which the soil was allowed to drain freely for approximately 30 min until no more water dripped out. The mass of the wet soil was recorded, and the water-holding capacity was calculated as:

$$W_{cap}=M_{wet}-M_{dry}$$

where *W*_cap_ is the capillary water-holding capacity (g), *M*_wet_ is the mass of wet soil after free drainage (g), and *M*_dry_ is the mass of oven-dried soil (105 °C, 24 h) (g).

The measured capacity was 25 g of water per 100 g of oven-dried soil (i.e., a gravimetric water content of 25%), which served as the 100% RSWC baseline. Based on this baseline, six RSWC levels—0%, 20%, 40%, 60%, 80%, and 100%—were established by adding 0, 5, 10, 15, 20, and 25 g of distilled water, respectively, to 100 g of oven-dried soil. The soil was then sealed and allowed to equilibrate for 24 h before use. Throughout the experiment, soil moisture was maintained by daily weighing and replenishment of evaporated water with distilled water, keeping each treatment within ±2% of its target RSWC. Soil moisture content (%) was calculated as:

$$\mathrm{Soil}\;\mathrm{moisture}\;(\%)\;=\;\frac{\;\mathrm{Weight}\;\mathrm{of}\;\mathrm{added}\;\mathrm{distilled}\;\mathrm{water}}{\mathrm{Saturated}\;\mathrm{soil}\;\mathrm{mass}\;-\;\mathrm{Dry}\;\mathrm{soil}\;\mathrm{mass}}\;\times 100\%$$

### 2.4. Emergence and Pupal Survival Assays

Six soil moisture treatments were established, each with five replicate boxes. Each box contained twenty 1-day-old pupae buried at a depth of 1 cm, giving a total of 30 transparent plastic boxes (14 × 10 × 5 cm). The number of emerged adults, time to emergence, and morphological malformations (e.g., wing deformities) were recorded daily. To non-destructively monitor pupal survival, the pupae were placed against the inner wall of the transparent box at the same burial depth (1 cm), allowing direct visual inspection. The soil moisture in each treatment was kept constant throughout the experiment by daily gravimetric replenishment of water. Each box served as an experimental unit, with five replicate boxes per humidity treatment. Final emergence percentage was calculated per box and analyzed by one-way ANOVA, with humidity treatment as a fixed factor (df = 5, 24). Daily survival rate was also calculated on a per-box basis, and treatment means ± standard errors were plotted. All analyses were performed in SPSS (version 26.0) with a significance level of α = 0.05.

### 2.5. Moisture Preference Assay

A four-choice apparatus (10 × 10 × 6 cm box partitioned into four compartments) offered larvae simultaneous access to soils at 20%, 40%, 60% and 80% moisture. Fresh tomato leaves were placed at the center to provide feeding and a standardized starting point; twenty fourth-instar larvae were introduced per replicate on the central leaf. After 12 h intervals until all the larvae had pupated, the pupae were collected from each compartment and counted to calculate pupation preference percentage per moisture level. Seven replicates were conducted.

### 2.6. Pupation Depth Assay

Boxes (14 × 10 × 7 cm) with a 5 cm soil column were used to record the vertical distribution of pupation. Twenty late fourth-instar larvae were introduced into each box and allowed to burrow and pupate. After all the larvae had entered pupation, the soil was carefully excavated in 0.5 cm slices, and the pupae at each depth interval (0–0.5, 0.6–1.0, 1.1–1.5, and 1.6–2.0 cm) were counted. The experiment was replicated three times.

### 2.7. Statistical Analysis

All data were tested for normality and homogeneity of variances. One-way ANOVA was employed to compare final emergence rates among moisture treatments. For pupal survival analysis, the Kaplan–Meier method was used to estimate survival curves, and differences among treatments were compared using the log-rank test. Repeated-measures ANOVA (general linear model) was applied to daily survival rate data (moisture as the between-subject factor, time as the within-subject factor), pupation preference in the choice assay (moisture as the within-subject factor), and depth distribution within each moisture treatment (depth as the within-subject factor). The Greenhouse–Geisser correction was applied when Mauchly’s test of sphericity was significant. When ANOVA assumptions were met, Tukey’s HSD test was used for post hoc multiple comparisons (or Bonferroni correction for repeated-measures). Data analysis was performed using SPSS (version 26.0), and visualization was carried out with GraphPad Prism 11.0.2 and Origin 2024.

## 3. Results

### 3.1. Emergence Rates Across Moisture Gradients

**Figure 1:**
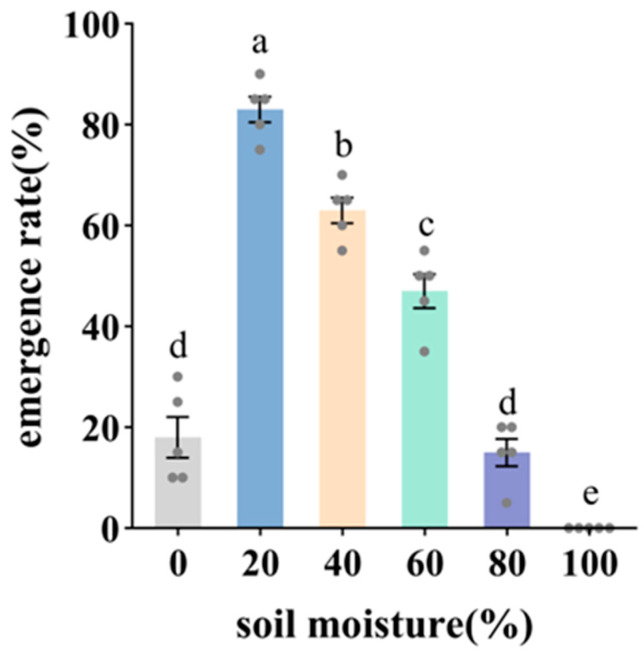
The emergence rate of *Tuta absoluta* under different soil moisture levels. The data are presented as mean ± standard error. Different lowercase letters above the bars indicate statistically significant differences among the soil moisture treatments according to Tukey’s HSD test (*p* < 0.05).

Soil moisture significantly affected emergence rates (one-way ANOVA: F_5,24_ = 126.27, *p* < 0.001; Figure 1). The data are presented as mean ± SEM. The highest emergence rate was recorded at 20% soil moisture (83 ± 2.55%), which was significantly greater than all other treatments. Under 40% and 60% moisture, emergence decreased to 63 ± 2.55% and 47 ± 3.39%, respectively. Emergence was strongly suppressed at 0% (18 ± 4.06%) and 80% (15 ± 2.73%) moisture, and no adults emerged at 100% moisture (0% emergence within two days). These outcomes demonstrate a unimodal response with an optimum at moderately dry moisture levels (20–40%).

### 3.2. Pupal Survival Dynamics

**Figure 2:**
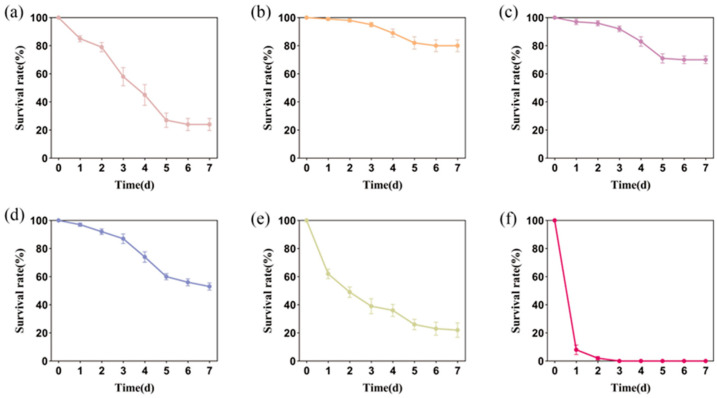
The survival rate dynamics of *Tuta absoluta* (Meyrick) pupae under different soil moisture conditions. Subfigures (**a**), (**b**), (**c**), (**d**), (**e**), and (**f**) correspond to the treatment groups with soil moisture levels of 0%, 20%, 40%, 60%, 80%, and 100%, respectively. The vertical axis represents the survival rate (%) of the *Tuta absoluta* pupae, and the horizontal axis represents the incubation time (**d**) after treatment. Error bars in the figure indicate the standard error of the mean from multiple experimental replicates.

Kaplan-Meier survival analysis showed that soil moisture significantly affected pupal survival time (log-rank test: χ^2^ = 461.4, df = 5, *p* < 0.0001; Figure S1). Daily survival trajectories are presented in Figure 2. Throughout the observation period, the 20% moisture group maintained the highest survival rate, declining gradually from day 4 and ending at 80 ± 4.18% (Figure 2b; repeated-measures ANOVA, time effect: F_7,28_ = 18.69, *p* < 0.001). The 40% moisture group had a slightly lower survival rate, ending at 70 ± 2.74% (Figure 2c; F_7,28_ = 43.53, *p* < 0.001). The 60% moisture group showed a steady decline over time, with a final survival rate of 53 ± 2.55% (Figure 2d; F_7,28_ = 79.62, *p* < 0.001). The 80% moisture group declined rapidly from days 1 to 5 and eventually leveled off at 22 ± 5.15% (Figure 2e; F_7,28_ = 66.89, *p* < 0.001). The 100% moisture group experienced catastrophic mortality (all pupae died by day 3) (Figure 2f). The 0% treatment group exhibited a progressive decline, with a final survival rate of 24 ± 4.30% (Figure 2a; F_7,28_ = 131.53, *p* < 0.001). These survival trajectories indicate strong moisture-dependent mortality risks at both extremes of the moisture gradient.

### 3.3. Pupation Moisture Preference

**Figure 3:**
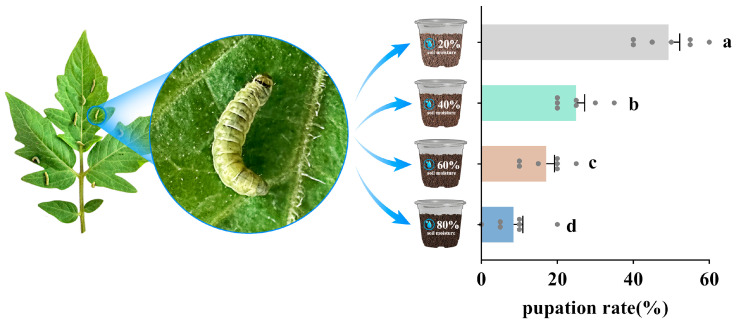
The pupation rates of *Tuta absoluta* larvae reared under different soil moisture levels (20%, 40%, 60%, 80%). Different lowercase letters to the right of the bars indicate significant differences among soil moisture treatments according to Tukey’s HSD test (*p* < 0.05).

In the choice assay offering 20%, 40%, 60% and 80% soil moistures, the larvae preferentially pupated in the 20% soil compartment (mean pupation percentage ± SE = 49.29 ± 2.97%), which was significantly higher than the pupation percentages for other moisture levels (repeated-measures ANOVA: F_3,18_ = 38.7, *p* < 0.001; Figure 3). Pupation percentages in the 40%, 60% and 80% compartments were 25.00 ± 2.18%, 17.14 ± 2.14% and 8.57 ± 2.37%, respectively. Thus, larvae show an active behavioral selection for relatively low soil moisture when given a simultaneous choice.

### 3.4. Pupation Depth Distribution

**Figure 4:**
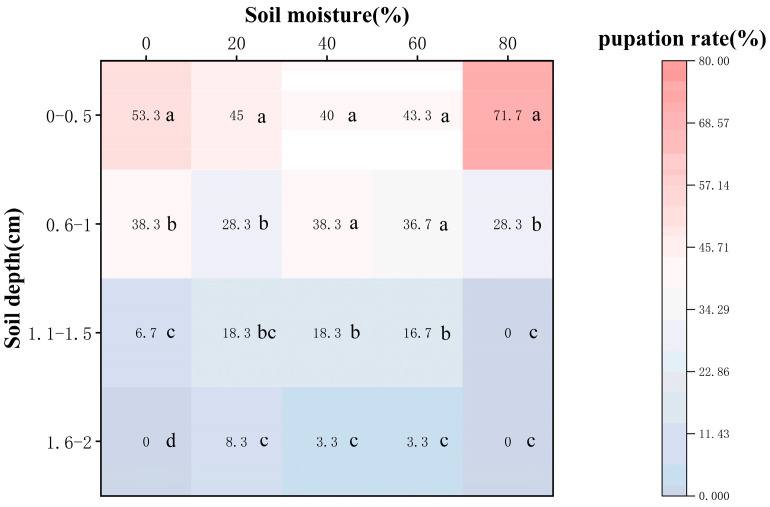
A heat map of the tomato leafminer pupation rate under different soil moisture and soil depth combinations. The horizontal axis represents soil moisture (%), and the vertical axis represents soil depth (cm). The values in the figure represent the pupation rate (%) of the tomato leafminer, with the color gradient on the right corresponding to the pupation rate (darker colors indicate higher pupation rates). Under the same soil moisture or soil depth conditions, different lowercase letters following the values indicate significant differences in pupation rates among the groups (*p* < 0.05).

The pupae were predominantly located in the upper soil strata (0–1 cm), and their depth distribution was influenced by soil moisture (Figure 4). Under 0% moisture, pupation differed significantly among depth layers (repeated-measures ANOVA, F_3,6_ = 59.47, *p* = 0.009), with the pupae concentrated near the surface (0–0.5 cm and 0.6–1.0 cm). Under 20% moisture, depth distribution did not differ significantly (F_3,6_ = 14.67, *p* = 0.051), and the pupae were still distributed across shallow depths but concentrated near the surface. At 40% moisture, depth distribution differed significantly (F_3,6_ = 55.00, *p* = 0.005), with the 0–0.5 cm layer (40 ± 2.89%) and the 0.6–1.0 cm layer (38.8 ± 1.67%) sharing similar proportions. At 60% moisture, depth distribution also differed significantly (F_3,6_ = 36.40, *p* = 0.019), with the 0–0.5 cm layer (43.3 ± 1.67%) and the 0.6–1.0 cm layer (36.7 ± 4.41%) again accounting for the majority of pupae. Under 80% moisture, depth distribution was highly significant (F_3,6_ = 154.75, *p* = 0.006), with pupation strongly concentrated in the shallowest layer (0–0.5 cm; 71.7 ± 3.33%) and no pupae recovered from deeper strata. These patterns indicate a tendency for larvae to pupate nearer the surface and for higher moisture to restrict pupation to very shallow microhabitats.

## 4. Discussion

Our results show a strong, non-linear (unimodal) relationship between soil moisture and pupal success in *T. absoluta*: moderate dryness (20–40%) maximized pupation preference, survival and emergence. Such moisture-dependent effects are widely observed in other insects, including *Spodoptera exigua* [10], *S. frugiperda* [9,11], *Helicoverpa zea* [12,13], and *Contarinia nasturtii* [14]. For instance, soil saturation (>88%) can completely block the normal pupation of *S. frugiperda*, while the pupal survival of *H. zea* declines markedly in compacted soils [13]. Additionally, in the present study, both extreme desiccation and saturation produced high mortality. Similar patterns have been reported for other Lepidoptera such as the corn earworm [15] and the striped cutworm [7], where irrigation or rainfall sharply reduces emergence rates, as well as for the rose leaf beetle [8], a Hymenoptera pest displaying comparable biology.

This pattern is consistent with findings across diverse soil-dwelling insects. In Lepidoptera, *Ectropis grisescens* showed significantly reduced emergence in dry (20%) or wet (80%) sandy loam compared to intermediate levels, and pupae buried with 80–moisture soil exhibited lower emergence success than unburied individuals [16,17]; *Heortia vitessoides* exhibited suppressed burrowing and emergence under extremely dry (0%) or saturated (80–100%) conditions [18]; and *Spodoptera frugiperda* emergence dropped to zero after 72 h of continuous saturated soil treatment [19]. Comparable responses are documented in Diptera: *Bactrocera tryoni* experienced 85% pupal mortality at 0% soil moisture and 30% at 100% moisture, with minimal losses at intermediate levels [20]; *B. dorsalis* emergence exceeded 90% at 10–60% moisture but fell to zero at 0%, 80%, 90%, and 100% moisture [21,22]; and *Megaselia scalaris* and *Dohrniphora cornuta* developed optimally at 20–40% moisture across three soil types [23]. Coleopteran species show similar sensitivity: *Aethina tumida* larvae in dry/tilled soil died within 9 days without burrowing, while those in wet soil successfully pupated [24]. High soil moisture also drives mortality in *Pectinophora gossypiella*, where saturated soil conditions (>195% gravimetric moisture) at 10 °C caused approximately 60% mortality of diapausing larvae within 10 days [25]. The broad phylogenetic distribution of these moisture-dependent patterns—spanning an estimated 25% of Lepidoptera species that interact with soil [26]—suggests that extreme soil moisture is a common constraint on pupal survival, with *T. absoluta* appearing particularly sensitive to saturation.

Behaviorally, *T. absoluta* larvae preferred relatively dry microhabitats (20% moisture) and consistently pupated within the top 1 cm of soil. This shallow pupation aligns with depth distributions reported for other insects: *Anastrepha* spp. larvae typically burrowed no more than 2–5 cm before pupating [27]; *B. oleae* larvae predominantly pupated in the top 3 cm, with mean depth varying significantly by soil type and moisture (greater at 50% than at 10% field capacity) [28]; and *Rhagoletis mendax* depth was significantly affected by mulch type and moisture, with approximately 50% of individuals pupating on the surface of wet soil [29]. The tendency of *T. absoluta* to shift pupation even shallower under high moisture (80% soil moisture) was also observed in *B. dorsalis*, where more than 50% of larvae pupated on the soil surface at 80–100% moisture, compared to none at 0–70% moisture [21,22], and in *H. vitessoides*, which avoided burrowing into saturated substrates [18]. Notably, the 20% treatment was the only condition where depth distribution was non-significant, suggesting behavioral flexibility at this optimal moisture level.

The mechanisms underlying moisture-dependent mortality were not directly measured in the present study, but the literature suggests several factors. Soil saturation can reduce oxygen availability in pore spaces [19], while dry soils may cause desiccation stress that reduces pupal body water content and emergence success, as demonstrated in *E. grisescens* where 20–moisture sandy loam significantly decreased pupal water content [16]. Soil physical properties modulate these effects: *Heliothis zea* emergence decreased significantly with increasing soil compaction, with excavation confirming that compaction rather than physical damage to pupae was the primary cause [30]; *B. dorsalis* showed different pupation depth and survival patterns across sandy loam to sandy clay soils [21,22]; *B. oleae* depth was influenced by temperature–soil type and soil type–moisture interactions [28]; *L. sericata* development time, pupal weight, and adult weight were significantly affected by soil type × moisture interactions [31]; and *S. frugiperda* pupae from initially dry soil survived saturation stress better than those from moist soil [19]. For phorid flies, soil moisture was the most influential factor, with 20–40% moisture being optimal across loamy sand and sandy loam [23]. Soil surface conditions can independently drive mortality: *S. sorghicola* larvae diapausing on the soil surface suffered severe mortality from high surface temperatures (>40 °C), whereas those buried at 5–10 cm emerged consistently [32].The substantial mortality of *P. gossypiella* under saturated conditions further demonstrates that excessive soil moisture alone, without invoking biotic agents, can be a significant mortality factor for soil-dwelling lepidopteran pupae [25]. Because only a single sandy loam was used, the moisture thresholds identified here may shift under different soil textures.

Several limitations should be noted. The experiments were conducted under controlled laboratory conditions with a single soil type, and measurements of oxygen levels or physiological stress markers were not performed. The phylogenetic breadth of soil-insect associations—particularly in Lepidoptera [26]—highlights the general importance of these interactions, yet species-specific responses vary considerably, as shown by the diverse moisture preferences across *B. dorsalis* [21,22], *B. oleae* [28], *A. tumida* [24], phorid flies [23], *H. vitessoides* [18], *R. mendax* [29], *R. indifferens* [33], *L. sericata* [31], *P. gossypiella* [25], and *S. frugiperda* [19]. Future work should prioritize field validation across soil types and climates, physiological investigation of moisture-dependent mortality mechanisms, and evaluation of whether short-duration moisture pulses can achieve population suppression without adverse agronomic consequences.

## 5. Conclusions

This study quantitatively demonstrates for the first time how soil moisture influences key biological parameters during the pupal stage of *T. absoluta*. Our laboratory assays establish that soil moisture is a decisive abiotic filter for *T. absoluta* pupation behavior and demographic success: moderate dryness (20–40%) optimizes pupation preference and emergence, while both desiccation and high saturation impose high pupal mortality. Pupation occurs predominantly in the uppermost soil strata (0–1 cm), making the immediate surface a tractable control target. These moisture-dependent vulnerabilities create opportunities for practical IPM and can be engineered to increase mortality at the pupal stage while minimizing chemical inputs. Overall, exploiting the soil-phase ecology of *T. absoluta* offers a promising pathway to sustainable, ecology-based pest suppression.

## Data Availability

The original contributions presented in this study are included in the article. Further inquiries can be directed to the corresponding author.

## Supplementary Materials

The following supporting information can be downloaded at https://www.mdpi.com/article/10.3390/insects17060603/s1, Figure S1: Kaplan–Meier survival curves of *Tuta absoluta* pupae under six soil moisture treatments (0%, 20%, 40%, 60%, 80%, and 100%).

## References

[1] Desneux N., Desneux N., Wajnberg E., Wyckhuys K.A.G., Burgio G., Arpaia S., Narváez-Vasquez C.A., González-Cabrera J., Catalán Ruescas D., Tabone E. (2010). Biological invasion of European tomato crops by *Tuta absoluta*: Ecology, geographic expansion and prospects for biological control. J. Pest Sci..

[2] Bawin T., De Backer L., Dujeu D., Legrand P., Megido R.C., Francis F., Verheggen F.J. (2014). Infestation Level Influences Oviposition Site Selection in the Tomato Leafminer *Tuta absoluta* (Lepidoptera: Gelechiidae). Insects.

[3] Chen L., Li X., Zhang J., He T., Huang J., Zhang Z., Wang Y., Hafeez M., Zhou S., Ren X. (2021). Comprehensive Metabolome and Volatilome Analyses in Eggplant and Tomato Reveal Their Differential Responses to *Tuta absoluta* Infestation. Front. Plant Sci..

[4] Cuthbertson A.G., Mathers J.J., Blackburn L.F., Korycinska A., Luo W., Jacobson R.J., Northing P. (2013). Population development of *Tuta absoluta* (Meyrick) (Lepidoptera: Gelechiidae) under simulated UK glasshouse conditions. Insects.

[5] Biondi A., Guedes R.N.C., Wan F.H., Desneux N. (2018). Ecology, worldwide spread, and management of the invasive South American tomato pinworm, *Tuta absoluta*: Past, present, and future. Annu. Rev. Entomol..

[6] Mawcha K.T., Kinyanjui G., Berhe D.H., Hategekimana A., Joelle K., Ndolo D. (2025). An overview of sustainable management strategies for *Tuta absoluta*. Int. J. Pest Manag..

[7] Murray D.A.H., Zalucki M.P. (1990). Effect of soil moisture and simulated rainfall on pupal survival and moth emergence of *Helicoverpa punctigera* (Wallengren) and *H. armigera* (Hübner) (Lepidoptera: Noctuidae). Aust. J. Entomol..

[8] Cui J., Yin J., Dong L., Gao Y., Shi S., Zou J., Li W., Wang Y. (2025). Impact of Temperature and Soil Moisture on the Life Cycle of the Strawberry Pest *Priophorus fulvostigmatus* and Its Control. Insects.

[9] He L., Zhao S., Ali A., Ge S., Wu K. (2021). Ambient humidity affects development, survival, and reproduction of the invasive fall armyworm, *Spodoptera frugiperda* (Lepidoptera: Noctuidae), in China. J. Econ. Entomol..

[10] Zheng X.L., Wang P., Lei C.L., Lu W., Xian Z.H., Wang X.P. (2013). Effect of soil moisture on overwintering pupae in *Spodoptera exigua* (Lepidoptera: Noctuidae). Appl. Entomol. Zool..

[11] Shi Y., Li L.Y., Shahid S., Smagghe G., Liu T.X. (2021). Effect of soil moisture on pupation behavior and inhabitation of *Spodoptera frugiperda* (Lepidoptera: Noctuidae). Appl. Entomol. Zool..

[12] Schardong I.S., Reisig D.D., Possebom T., Heitman J. (2024). *Helicoverpa zea* Boddie (Lepidoptera: Noctuidae) pupal success and adult eclosion across variable soil type and moisture. Environ. Entomol..

[13] Dillard D., Reisig D.D., Schug H.T., Burrack H.J. (2023). Moisture and soil type are primary drivers of *Helicoverpa zea* (Lepidoptera: Noctuidae) pupation. Environ. Entomol..

[14] Chen M., Shelton A.M. (2014). Impact of soil type, moisture, and depth on swede midge (Diptera: Cecidomyiidae) pupation and emergence. Environ. Entomol..

[15] Yu F.L., Wu G., Liu T.J., Zhai B.P., Chen F.J. (2008). Effects of irrigation on the performance of cotton bollworm, *Helicoverpa armigera* (Hübner) during different pupal stages. Int. J. Pest Manag..

[16] Wang H., Ma T., Xiao Q., Cao P., Chen X., Wen Y., Xiong H., Qin W., Liang S., Jian S. (2017). Pupation behaviors and emergence successes of *Ectropis grisescens* (Lepidoptera: Geometridae) in response to different substrate types and moisture contents. Environ. Entomol..

[17] Wang C., Wang H.F., Ma T., Xiao Q., Cao P.R., Chen X., Xiong H.P., Qin W.Q., Sun Z.H., Wen X.J. (2018). Choice and no-choice bioassays to study the pupation preference and emergence success of *Ectropis grisescens*. J. Vis. Exp..

[18] Wen Y., Jin X., Zhu C., Chen X., Ma T., Zhang S., Zhang Y., Zeng S., Chen X., Sun Z. (2016). Effect of substrate type and moisture on pupation and emergence of *Heortia vitessoides* (Lepidoptera: Crambidae): Choice and no-choice studies. J. Insect Behav..

[19] Tian T., Zhai Y., Chen Z., Yang Y., Hong B. (2025). Effects of saturated soil moisture on fall armyworm pupal development. Insects.

[20] Hulthen A.D., Clarke A.R. (2006). The influence of soil type and moisture on pupal survival of *Bactrocera tryoni* (Froggatt) (Diptera: Tephritidae). Aust. J. Entomol..

[21] Bilal M., Mamoon-ur-Rashid M., Wazir S., Naeem M., Ishaq A., Ullah R.M.K., Aldawood A.S. (2025). Effect of soil types and moisture contents on the pupation behavior and adult emergence of Oriental fruit fly, *Bactrocera dorsalis* (Hendel) (Diptera: Tephritidae). Int. J. Trop. Insect Sci..

[22] Hou B., Xie Q., Zhang R. (2006). Depth of pupation and survival of the Oriental fruit fly, *Bactrocera dorsalis* (Diptera: Tephritidae) pupae at selected soil moistures. Appl. Entomol. Zool..

[23] Han W., Feng D., Tang Y. (2024). The effect of soil type and moisture on the development of forensically important *Megaselia scalaris* and *Dohrniphora cornuta* (Diptera: Phoridae). Insects.

[24] Ellis J.D., Hepburn R., Luckman B., Elzen P.J. (2004). Effects of soil type, moisture, and density on pupation success of *Aethina tumida* (Coleoptera: Nitidulidae). Environ. Entomol..

[25] Venette R.C., Naranjo S.E., Hutchison W.D. (2000). Implications of larval mortality at low temperatures and high soil moistures for establishment of pink bollworm (Lepidoptera: Gelechiidae) in southeastern United States cotton. Environ. Entomol..

[26] Legal L. (2023). “Lepidoptera Flies”, but Not Always…Interactions of Caterpillars and Chrysalis with Soil. Diversity.

[27] Hodgson P.J., Sivinski J., Quintero G., Aluja M. (1998). Depth of pupation and survival of fruit fly (*Anastrepha* spp.: Tephritidae) pupae in a range of agricultural habitats. Environ. Entomol..

[28] Dimou I., Koutsikopoulos C., Economopoulos A.P., Lykakis J. (2003). Depth of pupation of the wild olive fruit fly, *Bactrocera* (*Dacus*) *oleae* (Gmel.) (Dipt., Tephritidae), as affected by soil abiotic factors. J. Appl. Entomol..

[29] Renkema J.M., Cutler G.C., Lynch D.H., MacKenzie K., Walde S.J. (2011). Mulch type and moisture level affect pupation depth of *Rhagoletis mendax* Curran (Diptera: Tephritidae) in the laboratory. J. Pest Sci..

[30] Roach S.H., Campbell R.B. (1983). Effects of soil compaction on bollworm (Lepidoptera: Noctuidae) moth emergence. Environ. Entomol..

[31] Kökdener M., Şahin Yurtgan M. (2022). The effect of soil type and moisture level on the development of *Lucilia sericata* (Diptera: Calliphoridae). J. Med. Entomol..

[32] Franzmann B.A., Lloyd R.J., Zalucki M.P. (2006). Effect of soil burial depth and wetting on mortality of diapausing larvae and patterns of post-diapause adult emergence of sorghum midge, *Stenodiplosis sorghicola* (Coquillett) (Diptera: Cecidomyiidae). Aust. J. Entomol..

[33] Yee W.L. (2013). Soil moisture and relative humidity effects during postdiapause on the emergence of western cherry fruit fly (Diptera: Tephritidae). Can. Entomol..

