# Supplementary material for: The Effects of Soil Moisture on the Pupation, Survival, and Emergence of the Tomato Leafminer, Tuta absoluta
Source: Insects. 2026 Jun 8;17(6):603. doi: 10.3390/insects17060603 (PMC13299490; doi:10.3390/insects17060603)
Supplement: Supplementary file 1 [file insects-17-00603-s001.zip › insects-4299549-supplementary.pdf]

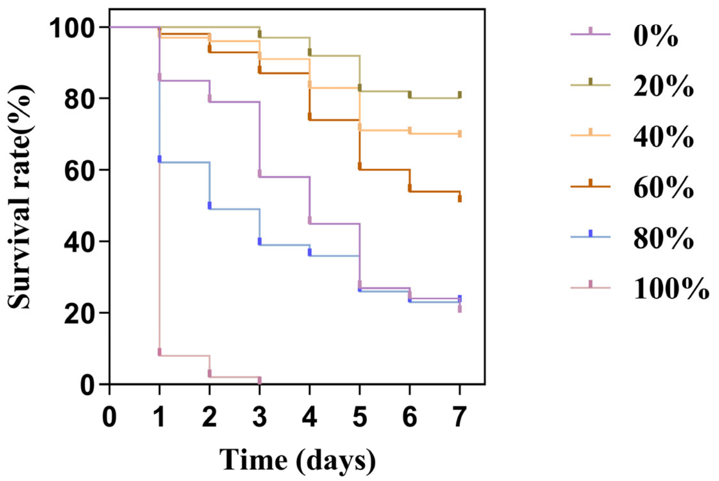

Figure S1. Kaplan–Meier survival curves of Tuta absoluta pupae under six soil moisture treatments (0%, 20%, 40%, 60%, 80%, and 100%). Each line represents a different moisture level. The vertical axis represents survival rate (%), and the horizontal axis represents time (days). Survival differed significantly among treatments (log-rank test: $\chi^2 = 461.4$, df = 5, $P < 0.0001$). Data are based on 100 pupae per treatment (20 pupae × 5 replicate boxes).
